# Use of Lichens to Evaluate the Impact of Post-Earthquake Reconstruction Activities on Air Quality: A Case Study from the City of L’Aquila

**DOI:** 10.3390/biology11081199

**Published:** 2022-08-10

**Authors:** Letizia Di Biase, Paolo Di Lisio, Loretta Pace, Lorenzo Arrizza, Simone Fattorini

**Affiliations:** 1 Department of Life, Health and Environmental Sciences, University of L’Aquila, Via Vetoio, 67100 L’Aquila, Italy; 2 Department of Physical and Chemical Sciences, University of L’Aquila, Via Vetoio, 67100 L’Aquila, Italy

**Keywords:** biomonitoring, cities, demolition, ecological indicators, Italy, lichen diversity value, lichens, pollution, urban ecology, urban–rural gradient

## Abstract

**Simple Summary:**

Lichens are a symbiotic association of fungi and algae. As few lichen species can tolerate high levels of pollution, they are widely used for air-quality monitoring. In this study, we used the Lichen Diversity Value (LDV), an index based on the diversity of lichens living on trees, to evaluate the effects of the reconstruction activities occurring in the city of L’Aquila after the 2009 earthquake that largely destroyed the city centre. We tested if the values of the LDV index changed along the urban–rural gradient in response to the presence of air pollutants produced by reconstruction works. We also used a rapid analytical technique (Energy-Dispersive X-ray Spectroscopy—EDS) to detect the main pollutants accumulated in the lichens. We found that the LDVs increased from the city centre towards suburban areas. The EDS analysis revealed a massive presence of aluminium and silicon (used in the manufacture of concrete) in the more central areas. Our study suggests that the LDV index can be profitably used to monitor air quality in urban areas subject to building demolition and reconstruction, and that EDS may be applied to lichen samples for the rapid detection of the main pollutants associated with these activities.

**Abstract:**

Lichens are widely used as bioindicators of air quality because of their ability to absorb chemical pollutants. We used the Lichen Diversity Value (LDV) index to assess the effects of the urban reconstruction activities in the city of L’Aquila ten years after the 2009 earthquake on air quality. Sampling was conducted from the city centre (still mostly under reconstruction and closed to traffic) to suburban areas (where reconstruction is minimal). We tested if the LDV index varied with distance from the city centre because of the presence of air pollutants produced by reconstruction works. We also used Energy-Dispersive X-ray Spectroscopy (EDS) to detect the main pollutants accumulated in the sampled lichens. The LDV increased from the city centre towards suburban areas. EDS revealed high concentrations of pollutants related to demolition and reconstruction activities, such as aluminium and silicon (used in the manufacture of concrete), in the more central areas. These results suggest that the LDV index can be a useful tool to monitor air quality, even on a small scale, and in urban environments subject to building demolition and reconstruction. Moreover, EDS could represent a good preliminary analytical technique to identify the air pollutants associated with all of these activities.

## 1. Introduction

Lichens are a symbiotic association between a fungus, which absorbs water and minerals from the colonized surface, and one or more partners (algae or cyanobacteria), which contain chlorophyll and provide carbon compounds from photosynthesis [1,2,3,4,5]. This association forms a perennial and long-living organism that maintains the same morphology over time [6]. Lichens grow slowly and do not possess roots, stomas or a well-developed cuticle [1,2,4,7]. Thus, lichens depend entirely on the deposition of mineral nutrients on the surface they colonize. All of the elements and ions needed for growth, but also the contaminants present in the surrounding environment, are passively absorbed by the lichen’s surface year-round [7,8,9]. Most lichens are also characterized by a long lifespan, which allows for the continuous deposition of atmospheric pollutants, making them excellent organisms for long-term biomonitoring [5,6,9,10,11]. Owing to these biological properties, coupled with their widespread presence in a large variety of environments and the easiness and inexpensiveness of their sampling [12,13,14], lichens are among the most studied and used bioindicators of air quality worldwide [5,15,16,17]. The use of lichens is even recommended by official programs of pollution assessment in several countries [18,19].

As few lichen species can tolerate high levels of pollution, alterations in lichen assemblages, such as the gradual disappearance of a species over a certain time or from certain sites, are considered indicators of the deterioration of air quality. Comparing lichen assemblages from polluted sites with those of unaltered areas can provide useful information regarding the nature and severity of the anthropogenic disturbance, especially in urban environments [5,16,20,21]. Qualitative observations, simply based on species presence/absence, can be improved using more quantitative information obtained by calculating indices specifically devised for the use of lichens as biological indicators [14,16,22]. In addition, the analysis of the bioaccumulated substances absorbed and stored in the lichen thalli can provide further information about the air contaminants [16,23].

On 6 April 2009, a severe earthquake (6.3 on the moment magnitude scale) occurred in the area including the city of L’Aquila (Central Italy) and destroyed most of its historical centre, with 67% of buildings declared too damaged to be used [24]. Immediately after the earthquake, the city centre was forcibly closed to traffic, and residents moved from their homes to temporary housing units or new towns around the city [25,26]. At the same time, a program for the reconstruction of the city centre was developed, with the expansion of some of the pre-existing outskirts and suburbs. The ordinary road system was largely reorganized: new roads were built, old ones were expanded [26], and many houses and public buildings were reconstructed. The associated activities of demolition and reconstruction caused significant amounts of pollutants and dust to be generated by the building sites nearby, leading to a rapid decrease in air quality [27].

In the present study, we aimed to use epiphytic lichens as a tool to monitor local air quality degradation caused by building and demolition works ten years after the 2009 earthquake. For this purpose, we evaluated lichen assemblages (characterized by the Lichen Diversity Value index [10,20]) along an urbanization gradient, from the city centre (where most of the area is still under reconstruction and partially closed to traffic) to suburban areas with a reduced degree of reconstruction and a more rural environment. The almost complete absence of traffic in the city centre is an important characteristic of this study system, because, under ordinary conditions, traffic is the main source of air pollution in urban areas. Notably, previous observations in the city centre highlighted the almost complete absence of lichens, which ensures that the current presence of lichens, even in the city centre, is the result of a recent, post-earthquake colonization. In addition to the application of the Lichen Diversity Value index for the assessment of air quality, we investigated how different pollutants were accumulated along the gradient using Energy-Dispersive X-ray Spectroscopy (EDS), an analytical technique that allows for the rapid identification of the chemical elements (and their relative abundance) present in a sample.

Specifically, we tested the following hypotheses: (1) the Lichen Diversity Value index increased along the urban–rural gradient, with low values in city centre areas strongly impacted by reconstruction works; and (2) chemicals on lichen surfaces were indicative of pollutants related to demolition and reconstruction activities.

## 2. Materials and Methods

The study was conducted in the municipality of L’Aquila (Abruzzi Region; 70,000 people). The city of L’Aquila is located about 700 m above sea level, in the middle of the Aterno river valley. Although the total area of L’Aquila municipality is 473.91 km^2^, most of the territory is occupied by rural and natural areas, and the urban area is much smaller, covering less than 15 km^2^.

L’Aquila is surrounded by the Gran Sasso massif (with average elevations around 2000 m and the highest peak (Corno Grande) reaching 2912 m), the Sirente–Velino chain (another high-altitude massif, with the highest elevation (M. Velino) at 2487 m) and the mountain group of Monte Ocre–Monte Cagno (a short chain with the highest peak (M. Ocre) reaching 2204 m). L’Aquila has a temperate climate influenced by its high altitude, belonging to the Cfb (temperate oceanic climate) type according to the Köppen–Geiger classification [28,29]. The average annual temperature is 11.9 °C, with a minimum average annual temperature of 6.5 °C and a maximum average annual temperature of 17.3 °C; the annual precipitation is 713 mm (data for the period 1951–2000 [30]). From a bioclimatic point of view, L’Aquila belongs to the oceanic bioclimate [31] (Biondi e Baldoni 1995). More specifically, the phyoclimate of L’Aquila is represented by a transitional oceanic–semicontinental bioclimate with the subhumid hombrotype [32].

For large-scale monitoring programs, ANPA guidelines [33] suggest a sampling design based on a regular grid of primary and secondary units. Because of the small scale of our sampling and the constraints of the urban environment, the sampling sites could not be organized in a regular grid, but followed the urban development of the city. We chose nine sites along an urban–rural gradient of about 7.2 km from the city centre towards the more rural suburban areas, passing through areas with high population densities. Sites were selected at random, with the obvious constraint of the presence of trees. To reduce the influence of traffic as a pollution source as much as possible, we also avoided sites too close to very busy roads. The sites were numbered from 1 to 9 according to their distance from the city centre (corresponding to Site 1). The coordinates of the sites and their distance from the city centre are given in Table 1. The sites’ locations are shown in Figure S1. Landscape pictures showing the sites are provided in Figure S2.

Sites 1, 2, 4 and 5 were located in the historical centre, where a consistent number of buildings have been under restoration and traffic has been virtually absent from the occurrence of the earthquake to the sampling period. Site 3 was located in a liminal zone, between the city centre and the immediate outskirts. From site 6 to site 9, sampling was conducted in areas open to vehicular traffic. Sites 7 and 9, however, were characterized by limited anthropic disturbance, being located in countryside areas almost without paved roads, far from the city centre and scarcely or completely not affected by reconstruction works. Site 8 was close to a very busy highway. Sampling was conducted between December 2019 and February 2020.

Lichen sampling was conducted following the general guidelines suggested by ANPA [33]. At each site, epiphytic lichens were sampled from three random trees of the same species using 50 × 10 cm vertical grids, split up into five 10 cm × 10 cm squares. On each tree, we positioned four vertical grids, one for each cardinal point, at a height of 100 cm above the ground. We discarded trees with damaged trunks or irregularities on their surfaces. Trees were chosen to be as similar as possible in terms of trunk inclination and diameter. With regards to the species (Table 1), we ensured that we used species with similar bark characteristics (especially pH) among those present in the sampling sites as best as we could.

Then, we determined and counted the lichen species in each 10 cm × 10 cm square to calculate the lichen species frequencies, i.e., the number of squares in which a given species was found (maximum of 20 and minimum of 1). Lichens were identified in the field using a magnifying lens. Lichens that could not be identified with certainty in the field were collected and identified in the laboratory using a microscope. Identification was based on different keys [34,35,36] and re-checked with ITALIC 7.0 [37]. The nomenclature follows Nimis and Martellos [37].

For each sampled tree, a Lichen Diversity Value (LDVT) was calculated as the sum of the average frequencies (number of 10 cm × 10 cm squares occupied) of all lichen species at each cardinal point [38,39,40]. The lichen frequencies are given in Table S1.

Additionally, to express the naturalness of each site, we calculated an index of lichen diversity at the site level (LDVS) as the average of the three LDVTs of the same site [38], and used the following categories proposed by Nimis [20]:

- LDVS > 50: very high naturalness
- 41 < LDVS ≤ 50: high naturalness
- 31 < LDVS ≤ 40: average naturalness
- 21 < LDVS ≤ 30: low naturalness
- 11 < LDVS ≤ 20: average alteration
- 1 ≤ LDVS ≤ 10: high alteration
- LDVS < 1: very high alteration

We tested if the values of the LDVT and LDVS indices, as well as the number of species recorded at each site, increased with distance from the city centre as a consequence of the decrease in air pollution due to reconstruction works using one-tailed Spearman’s rank correlation tests with α = 0.05. Because of the peculiar characteristics of site 8 (which was far from the city centre, but close to very busy highways), correlations were calculated both including and excluding this site.

We investigated whether the species distribution was nested across sites, i.e., the degree to which lichen assemblages of sites with fewer species were subsets of successively larger assemblages. Nestedness can be viewed as the spatial outcome of a species pool being “filtered” by local (site-specific) environmental constraints, with each species’ distribution among sites determined by its ability to overcome the constraints [41]. Environmental gradients can generate nested subset patterns if the species with the broadest tolerance persist throughout the gradient, while others with more limited tolerance are restricted to one end of it [41]. To assess nestedness, we compiled a presence/absence matrix of species (rows) per site (columns) and measured the nestedness of this matrix using the spectral radius [42,43]. Significance was assessed with 100 null matrices using the “proportional row and column totals” algorithm to calculate the Z-value.

We also took samples of *Xanthoria parietina* (L.) Th. Fr. and analysed each sample with a Scanning Electron Microscope with Energy-Dispersive X-ray Spectroscopy (SEM/EDS) to detect the main elements accumulated on the lichen’s surface. We used this lichen species as it is very tolerant to air pollution, and it was found at all sites except for site 2 (where no lichen was found).

As we preferred to reduce the removal of lichens as much as possible, especially to not compromise future monitoring, we limited this analysis to a selection of sites representative of the gradient, and in which *Xanthoria parietina* was relatively abundant. For these reasons, this analysis was conducted only for five sites out of the nine sites investigated in this study.

For this analysis, a Zeiss Gemini 500 Scanning Electron Microscope was used, equipped with an Oxford Instruments Ultim Max detector for Energy-Dispersive X-ray Spectroscopy. From each sample of *Xanthoria parietina*, we took 7–11 measurements of the element percentages depending on the morphology of the observed lichen’s surface. Measurements from the same sample were averaged prior to analyses. Special attention was paid to searching for particles of asbestos. SEM/EDS analyses were conducted at the Microscopy Centre of L’Aquila University, which is included in the Ministry of Health’s list of laboratories qualified to carry out analyses on asbestos pursuant to Ministerial Decree 14/05/96 within the “2018–2019 Qualification Program of Asbestos Laboratories”.

To investigate the relationships between the percentages of detected elements and sites, Principal Components Analysis (PCA) was conducted with the average percentages of elements as variables and sites as objects. PCA was performed using a singular value decomposition approach.

Spearman’s rank correlations were calculated with the function cor.test in R 4.1.3 software, whereas PCA was conducted with the function prcomp [44]. Nestedness analysis was conducted using the software NeD [45,46].

## 3. Results

A total of seven species of lichens (Table S2) were recorded. The correlation between the number of lichen species found at each site and the distance from the city centre was significant (r_s_ = 0.661, *p* = 0.026).

**Figure 1:**
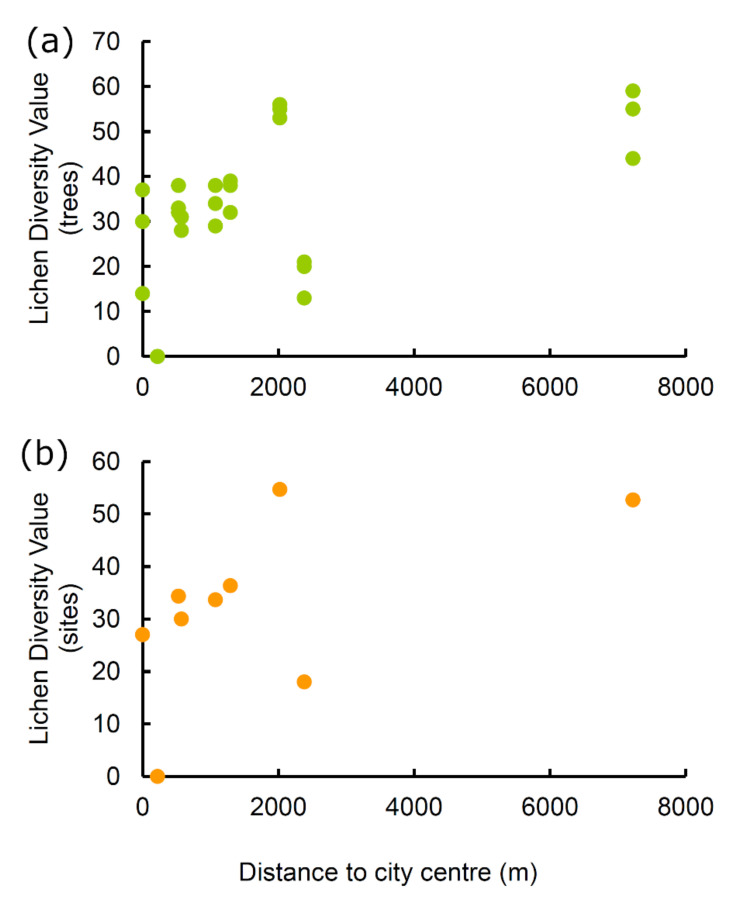
Relationship between Lichen Diversity Values and distance from the city centre along an urban–rural gradient in the city of L’Aquila (Central Italy). Lichen Diversity Values were calculated at: (**a**) tree level and (**b**) site level.

The Lichen Diversity Values at tree level (LDVTs) increased significantly with distance from the city centre (r_s_ = 0.505, *p* = 0.004) (Figure 1a). If site 8 (“Via Amiternum”) was omitted from the analysis, the correlation became even stronger (r_s_ = 0.790, *p* < 0.000001).

The Values of Naturalness (LDVSs) increased from the city centre to the rural areas, although the correlation was marginally non-significant (r_s_ = 0.533, *p* = 0.074) (Figure 1b, Table 1). If site 8 was omitted from the analysis, the correlation became significant (r_s_ = 0.881, *p* = 0.002).

Out of the nine sampling sites, only two (sites 7 and 9) reached the maximum level of environmental quality (very high naturalness); the remaining sites showed either average/low naturality (sites 1, 3, 4, 5 and 6) or average/very high alteration (sites 2 and 8) (Table 1).

The two sites with very high naturalness were characterized by the presence of all of the species found at the other sites. Overall, the species distribution across sites (Table S2) was significantly nested (Spectral Radius = 5.386, Z-score = 3, *p* < 0.001).

**Figure 2:**
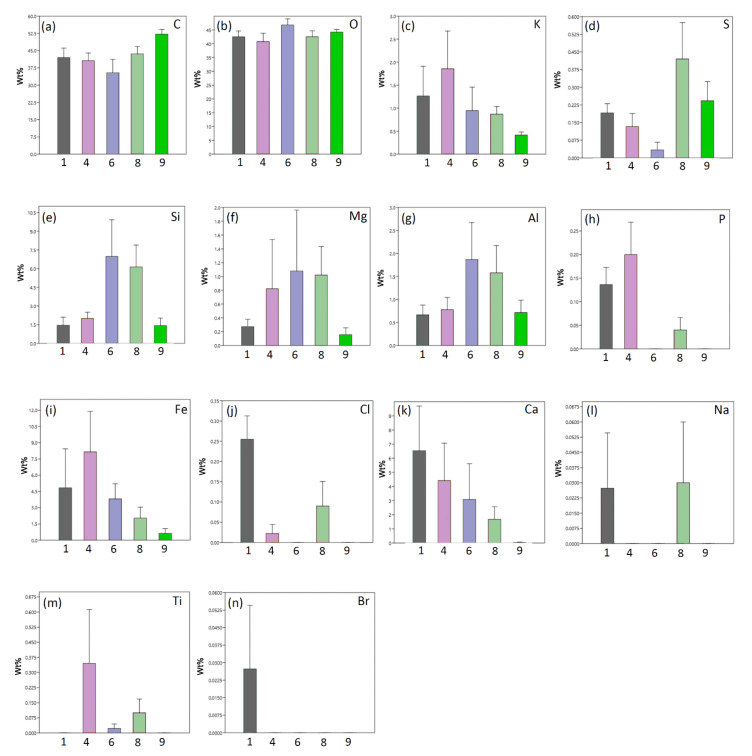
Averages weight percentages (Wt%) and standard errors of chemical elements found on samples of the lichen *Xanthoria parietina* obtained at sites along an urban–rural gradient in the city of L’Aquila (Central Italy): (**a**) carbonium; (**b**) oxygen; (**c**) potassium; (**d**) sulphur; (**e**) silicon; (**f**) magnesium; (**g**) aluminium; (**h**) phosphorus; (**i**) iron; (**j**) chlorine; (**k**) calcium; (**l**) sodium; (**m**) titanium; (**n**) bromine.

No particles of asbestos were found on the *Xanthoria parietina* samples examined by Scanning Electron Microscopy. The chemical analyses of the *Xanthoria parietina* samples through Energy-Dispersive X-ray Spectroscopy highlighted the presence of 14 elements (Figure S3, Table S3), with different proportions between sites. Most of them (C, O, K, S, Si, Mg, Al, Fe and Ca) were ubiquitous, although their proportions varied between sites (Figure 2). A few elements (P, Cl, Na, Ti and Br) were not detected at certain sites. C and O were the most abundant elements everywhere (Figure 2). C accounted for 35.4% to 52.1% of all detected elements (with an average of 42.7%). Similarly, O accounted for 40.8% to 46.8% of all detected elements (with an average of 43.3%). While C was distinctly more abundant at site 9, the values of O were more uniformly distributed (Figure 2). Ca and Fe were most abundant at Sites 1 and 4, while Si prevailed at Sites 6 and 8.

The PCA extracted five principal components, with the first two explaining more than 93% of the variance (Table 2).

The examination of the Principal Component loadings (Table 3) and the correlation coefficients between the original variables and the Principal Component scores (Table 4) indicated that the first component was positively correlated with the concentrations of Fe and Ca (and negatively with the concentration of C), whereas the second component was positively correlated with Si and, to a lesser extent, O.

**Figure 3:**
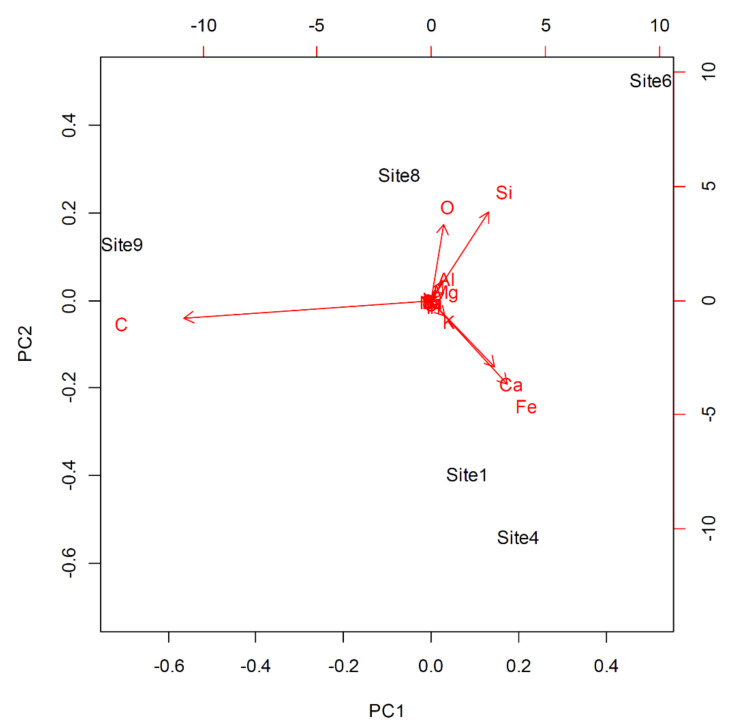
Biplot showing the position of samples of the lichen *Xanthoria parietina* collected along an urban–rural gradient in the city of L’Aquila (Central Italy) and analysed for the presence of chemical elements according to the results of Principal Components Analysis. The biplot shows the position of each sample in the space defined by the first two Principal Components (93.4% of variance). Arrows indicate the correlations between the Principal Components and element percentages.

Overall, the PCA results (Figure 3) indicated a distinct separation of sites located in the most interior part of the urban areas (influenced by high concentrations of Ca and Fe) from the others, which were mostly aligned along PC1, reflecting the urban–rural gradient. Site 6 was characterized by a high concentration of Si, while site 8 was characterized by O. Site 9, isolated at the other extreme of the gradient, was characterized by a high concentration of C.

## 4. Discussion

Lichens are among the most commonly used bioindicators for monitoring atmospheric quality because they include many species particularly sensitive to chemical pollution [47,48,49]. In particular, it is well known that air pollution in urban areas has detrimental effects on lichen diversity and abundance [50,51,52,53,54,55], to the point that lichens disappeared from most polluted cities in the 19th century [56,57].

The most common sources of pollution affecting the air quality of urban areas are industries, house heating and vehicular traffic [58,59,60,61]. Urban areas are, however, highly dynamic systems because they are subject to the continuous reshaping of buildings and infrastructure [58,60,62,63]. Urbanization, and particularly urban sprawl, imply not only the continuous construction of new buildings and streets, but also the demolition of former ones. These activities are obvious sources of air pollution [64,65,66,67]. In general, construction and demolition activities usually do not occur simultaneously across whole urban areas, but involve different sectors at different times, so their impacts might be relatively diluted. However, after catastrophic events (such as natural disasters or wars) that suddenly destroy large sectors of urban areas, reconstruction works will involve the virtually synchronous demolition and construction of large portions of the involved areas. Under such circumstances, massive amounts of pollutants are expected to be emitted to the atmosphere. This is the case of the city of L’Aquila.

L’Aquila was very severely damaged by an earthquake that occurred in 2009, which damaged thousands of buildings and rendered homeless around 30,000 people [68]. After more than ten years, large sectors of the city are still under reconstruction. Because of the demolition and reconstruction works, the city centre of L’Aquila has remained virtually inaccessible for this time. This has had two effects on air quality. While former sources of pollution, represented by vehicular traffic and house heating, virtually disappeared from many areas (especially the city centre), the same areas were obviously affected by the substances produced by the reconstruction works.

We found that lichens were very sensitive to these processes. While the disappearance of former sources of pollution allowed lichens to recolonize the city centre, the current reconstruction works have had profound impacts on the lichen assemblages. As our study is retrospective, we do not have specific data regarding the presence of lichens throughout the urban area of L’Aquila before the earthquake, but anecdotal observations conducted before the earthquake indicated their almost complete absence (L. Pace, personal observations).

In accordance with our first hypothesis that lichen biodiversity increases along the urban–rural gradient, we found that both the Lichen Diversity Values (LDVTs) and Values of Naturalness (LDVSs) increased from the city centre to the rural areas, a result also paralleled by the variation in lichen species richness. This pattern is consistent with previous research showing that lichen diversity increases along urban–rural gradients in response to increasing air quality [69,70,71]. Notably, we recorded the maximum levels of environmental quality at only two rural sites far from the city centre, whereas the air quality in the city centre was relatively poor.

According to a recent study based on lichen sampling on a regional scale, the environmental quality of the city of L’Aquila as a whole is between “high naturalness” and “average naturalness” [72]. Our study substantially confirms this general result, but highlights important differences between different sectors of the city.

Nestedness analysis revealed that the species distribution across sites was significantly nested, which indicated that lichens of assemblages with fewer species were subsamples of those with more species, and hence that the assemblages found in the city centre (in which only the most resistant species were present) tended to be subsamples of those of rural areas. Thus, the main effect of urban pollution is that of filtering species according to their ability to survive under increasing levels of air pollution. For example, *Xanthoria parietina, Physconia distorta* (With.) J.R. Laundon and *Physcia adscendens* H. Olivier, three species poorly tolerant to anthropic disturbance [73], were only found at suburban sites, while more tolerant species (*Evernia prunastri* (L.) Ach., *Melanelixia glabra* (Schaer.) O. Blanco, A. Crespo, Divakar, Essl., D. Hawksw. and Lumbsch and *Pleurosticta acetabulum* (Neck.) Elix and Lumbsch) [73] were widely distributed.

Interestingly, despite its not particularly high quality class, site 5 was characterized by the presence of the species *Parmelina tiliacea* (Hoffm.) Hale, *Melanelixia glabra* and *Pleurosticta acetabulum* (otherwise found only at the sites with the highest naturalness). This site was close to relatively large green spaces, which supports the importance of habitat characteristics in shaping lichen assemblages [74] and suggests a positive role of urban green spaces in ameliorating air quality.

The relatively poor quality of air in urban L’Aquila can only be associated with pollution from vehicular traffic in the case of site 8 (“Via Amiternum”), because all other sites were in places closed to traffic. The relatively low air quality in areas closed to traffic can be explained by the presence of pollutants from reconstruction activities, as shown by the chemical analyses.

Lichens are frequently used as ecological indicators in urban areas, especially to monitor the effects of pollutants produced by industrial activities and vehicular traffic [49,53,55,75]. However, to the best of our knowledge, our study is the first to involve the use of lichens to investigate the impact of reconstruction works.

In accordance with our second hypothesis that chemicals on lichen surfaces are indicative of pollution related to reconstruction works, the chemical analyses of samples of *Xanthoria parietina* showed the presence of certain elements associated with construction materials. Namely, the ordination analysis (PCA) clearly separated city centre sites (Sites 1 and 4), characterized by the presence of Ca and Fe, from more peripherical ones. CaO represents 63% by mass of Portland cement, and Fe is not only present in cement itself, but it is also the material of the rebars used in reinforced concrete. Fortunately, scanning electron microscopy imaging did not reveal the presence of asbestos. Asbestos is an important contaminant of urban air [76,77,78], and we were concerned about the possibility of high levels of asbestos emitted by demolition and reconstruction works. The absence of asbestos in our samples might be due to the fact that the majority of the most severely damaged buildings were constructed before the widespread use of asbestos.

## 5. Conclusions

Urban areas are subject to various forms of chemical pollution that decrease air quality with detrimental effects on the most sensitive organisms, such as lichens. We found that the reconstruction works following the earthquake that hit the city of L’Aquila had important effects on lichen assemblages. The traffic restrictions imposed by reconstruction works have allowed lichens to recolonize the city centre. However, at the same time, substances emitted into the atmosphere by reconstruction works represent a new source of air pollution with negative effects on lichens. Our study demonstrated the ability of lichens to capture these phenomena. Although catastrophic events, such as the earthquake of L’Aquila, are fortunately rare, urban areas are subject to continuous processes of construction and demolition, and air pollution associated with these activities should be taken into serious consideration, also for human health.

## Figures and Tables

**Table 1:** Geographic location, distance from city centre, Lichen Diversity Value and environmental quality of the nine sites investigated along the urban–rural gradient in the city of L’Aquila (Central Italy).

Site	Geographic Location	Distance from City Centre (Piazza Palazzo) (m)	Lichen Diversity Value (LDVS)	Environmental Quality	Sampled Tree
1 Piazza Palazzo	42.351185 N 13.398683 E	0	27.000	Low naturalness	*Tilia platyphyllos* Scop.
2 Piazza dei Nove Martiri	42.349842 N 13.400554 E	218	0.000	Very high alteration	*Quercus ilex* L.
3 Giovanni XXIII	42.351226 N 13.392293 E	527	34.333	Average naturalness	*Tilia platyphyllos* Scop.
4 Via dei Giardini	42.346035 N 13.398791 E	572	30.000	Low naturalness	*Cercis siliquastrum* L.
5 Via XXIV Maggio	42.341733 N 13.395884 E	1072	33.667	Average naturalness	*Tilia platyphyllos* Scop.
6 Via Colagrande	42.360944 N 13.406858 E	1293	36.333	Average naturalness	*Aesculus hippocastanum* L.
7 Via Mariana di Poggio di Roio	42.336272 N 13.384677 E	2022	54.667	Very high naturalness	*Quercus pubescens* Will.
8 Via Amiternum	42.366085 N 13.377949 E	2382	18.000	Average alteration	*Juglans regia* L.
9 Doline Monticchio/Ocre	42.312348 N 13.469104 E	7225	52.667	Very high naturalness	*Ostrya carpinifolia* Scop.

**Table 2:** Eigenvalues and percentages of explained variance for the Principal Components (PC) extracted for the distribution of chemical elements (in percentages) found on samples of the lichen *Xanthoria parietina* collected along an urban–rural gradient in the city of L’Aquila (Central Italy).

PC	Location Eigenvalue	Percentage of Explained Variance	Percentage of Cumulative Variance
1	45.091	69.448	69.448
2	15.552	23.953	93.401
3	2.85773	4.401	97.802
4	1.42676	2.198	100.000
5	4.30 × 10^−31^	6.62 × 10^−33^	100.000

**Table 3:** Loadings of the Principal Components extracted for the chemical elements (in percentages) found on samples of the lichen *Xanthoria parietina* collected along an urban–rural gradient in the city of L’Aquila (Central Italy).

Element	PC1	PC2	PC3	PC4	PC5
C	−0.903	−0.109	0.044	−0.028	−0.356
O	0.048	0.474	−0.675	0.441	−0.341
K	0.049	−0.100	0.082	0.046	−0.353
S	−0.012	0.003	0.044	−0.076	−0.275
Si	0.212	0.547	0.466	−0.295	−0.470
Mg	0.045	0.045	0.147	0.016	−0.216
Al	0.043	0.116	0.079	−0.034	0.113
P	0.004	−0.021	0.010	−0.002	−0.047
Fe	0.277	−0.519	0.251	0.568	−0.374
Cl	0.002	−0.012	−0.023	−0.074	−0.047
Ca	0.233	−0.410	−0.471	−0.615	−0.353
Na	0.000	0.000	0.001	−0.013	−0.013
Ti	0.006	−0.020	0.060	0.045	−0.082
Br	0.000	−0.002	−0.005	−0.006	−5.605 × 10^−5^

**Table 4:** Correlations for the Principal Components extracted for the chemical elements (in percentages) found on samples of the lichen *Xanthoria parietina* collected along an urban–rural gradient in the city of L’Aquila (Central Italy).

Element	PC1	PC2	PC3	PC4	PC5
C	−0.997	−0.071	0.012	−0.005	0.103
O	0.142	0.821	−0.501	0.232	0.267
K	0.614	−0.739	0.258	0.103	−0.406
S	−0.559	0.095	0.524	−0.635	0.147
Si	0.523	0.792	0.289	−0.129	−0.086
Mg	0.703	0.411	0.579	0.044	−0.473
Al	0.519	0.817	0.238	−0.072	0.087
P	0.299	−0.935	0.191	−0.020	−0.260
Fe	0.646	−0.711	0.147	0.236	−0.421
Cl	0.096	−0.446	−0.358	−0.815	0.755
Ca	0.627	−0.647	−0.319	−0.294	0.282
Na	0.001	−0.073	0.105	−0.992	0.605
Ti	0.275	−0.551	0.695	0.371	−0.839
Br	0.102	−0.492	−0.625	−0.597	0.787

## Data Availability

Original data are given in Table S1, Table S2 and Figure S3.

## Supplementary Materials

The following supporting information can be downloaded at: https://www.mdpi.com/article/10.3390/biology11081199/s1, Figure S1. Location of the sampling sites (map from Google Earth). The inset shows the position of the city of L’Aquila (red dot) within the Abruzzi Region (in blue); Figure S2: Street views of the nine investigated sites along the urban–rural gradient in the city of L’Aquila (Central Italy); Figure S3: Scanning Electron Microscope photographs and associated Energy-Dispersive X-ray Spectroscopy results for *Xanthoria parietina* samples collected at five sites along the urban–rural gradient in the city of L’Aquila (Central Italy). For each site, microphotographs and spectra of all examined samples are shown in sequence. Sites are numbered as in Table 1. Table S1: Number of lichen species recorded in each of the five quadrats of the vertical grids used to sample epiphytic lichens at nine sites (Sites, numbered from 1 to 9) in the city of L’Aquila (Central Italy). At each site, three trees (numbered from 1 to 3) were sampled, and the grid was placed at the four cardinal points. Site numbers as in Table 1. Table S2: Presence/absence of lichen species recorded at nine sites (Site, numbered from 1 to 9) in the city of L’Aquila (Central Italy). Table S3. Weight percentages of chemical elements found on samples of the lichen *Xanthoria parietina* sampled at five sites along an urban–rural gradient in the city of L’Aquila (Central Italy). Site numbers as in Table 1. Spectra are given in Figure S1.
