# Supplementary material for: Use of Lichens to Evaluate the Impact of Post-Earthquake Reconstruction Activities on Air Quality: A Case Study from the City of L’Aquila
Source: Biology (Basel). 2022 Aug 10;11(8):1199. doi: 10.3390/biology11081199 (PMC9405126; doi:10.3390/biology11081199)
Supplement: Supplementary file 1 [file biology-11-01199-s001.zip › SupplementaryMaterials Corrected/Figure S2.pdf]

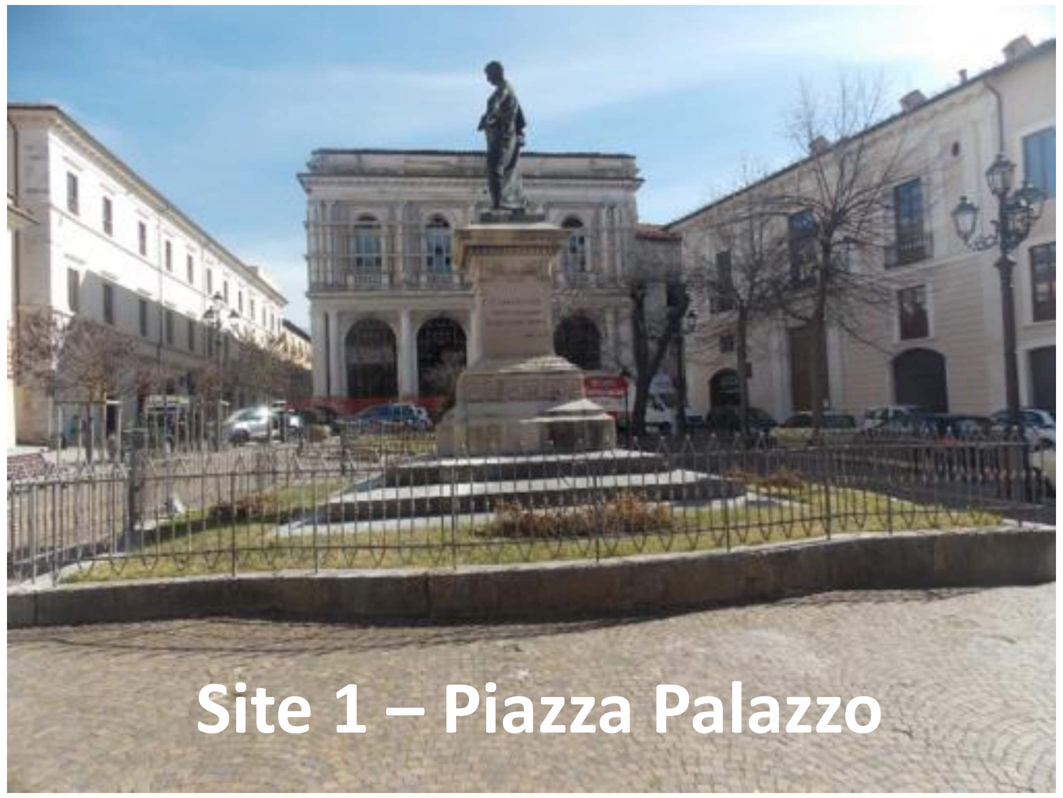
Site 1 – Piazza Palazzo

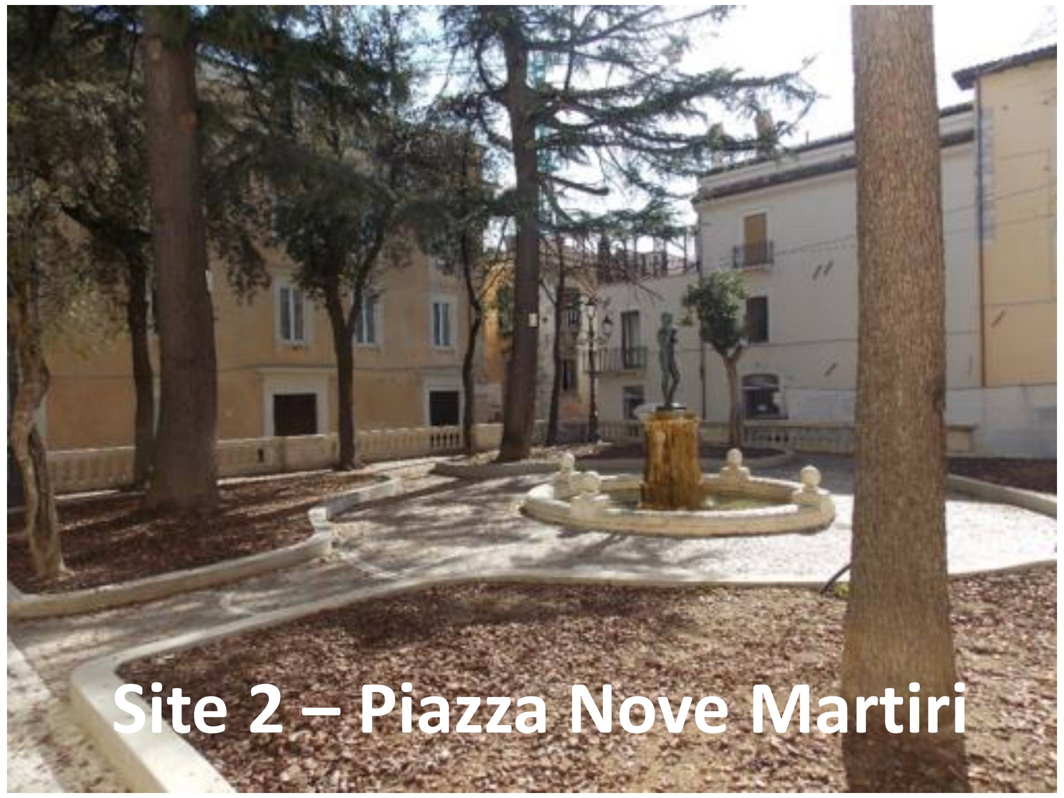
Site 2 – Piazza Nove Martiri

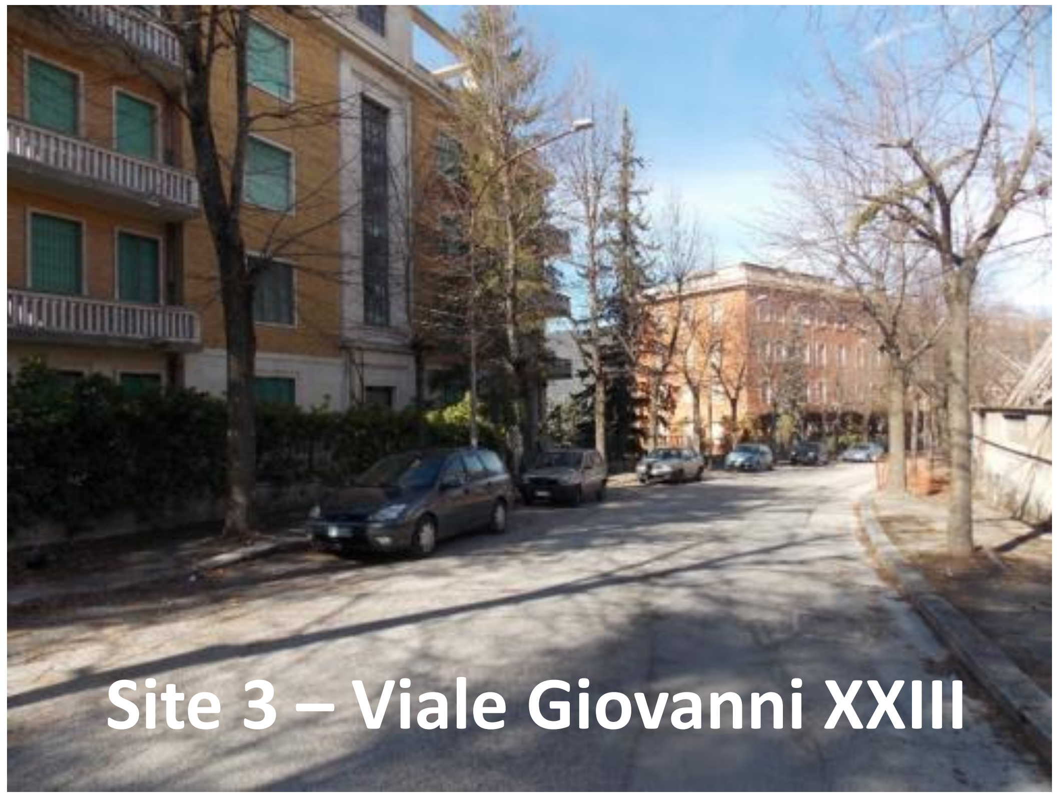
Site 3 – Viale Giovanni XXIII

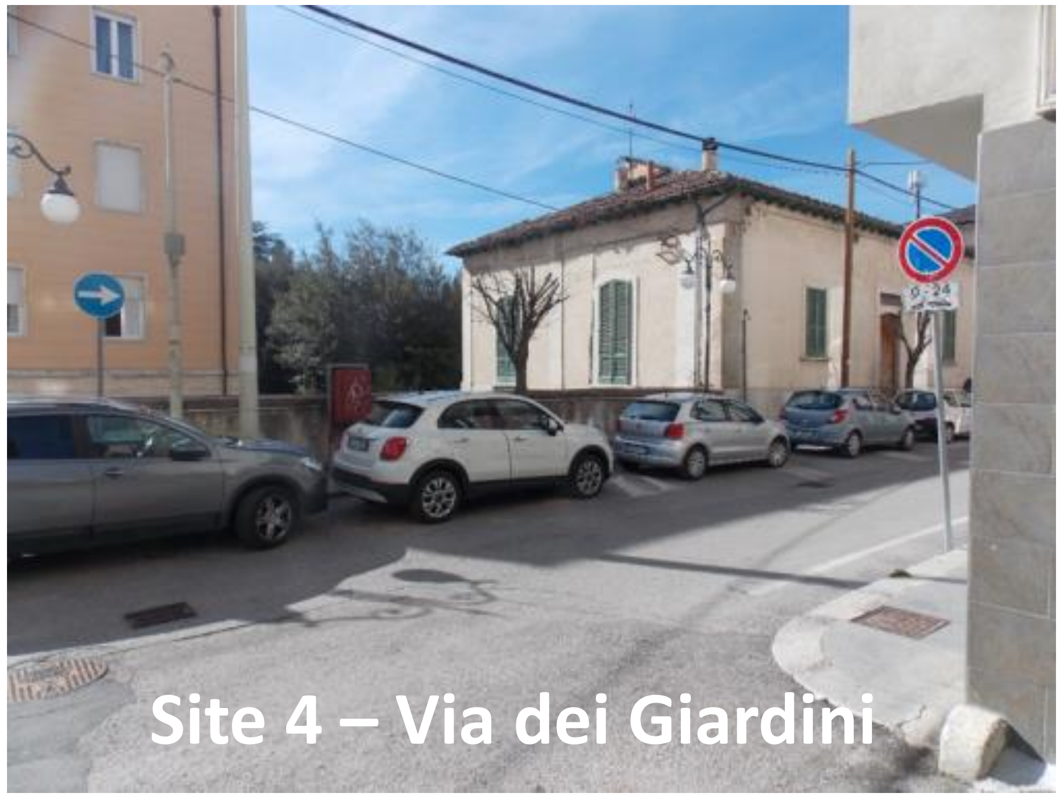
Site 4 – Via dei Giardini

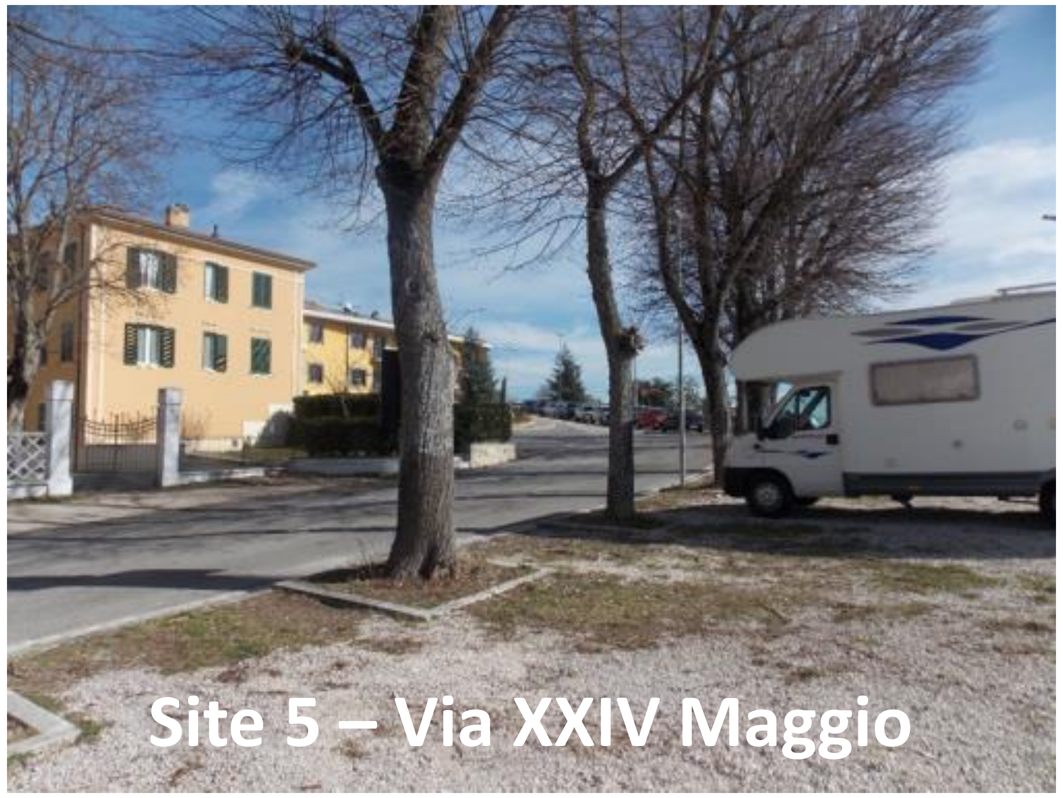
Site 5 – Via XXIV Maggio

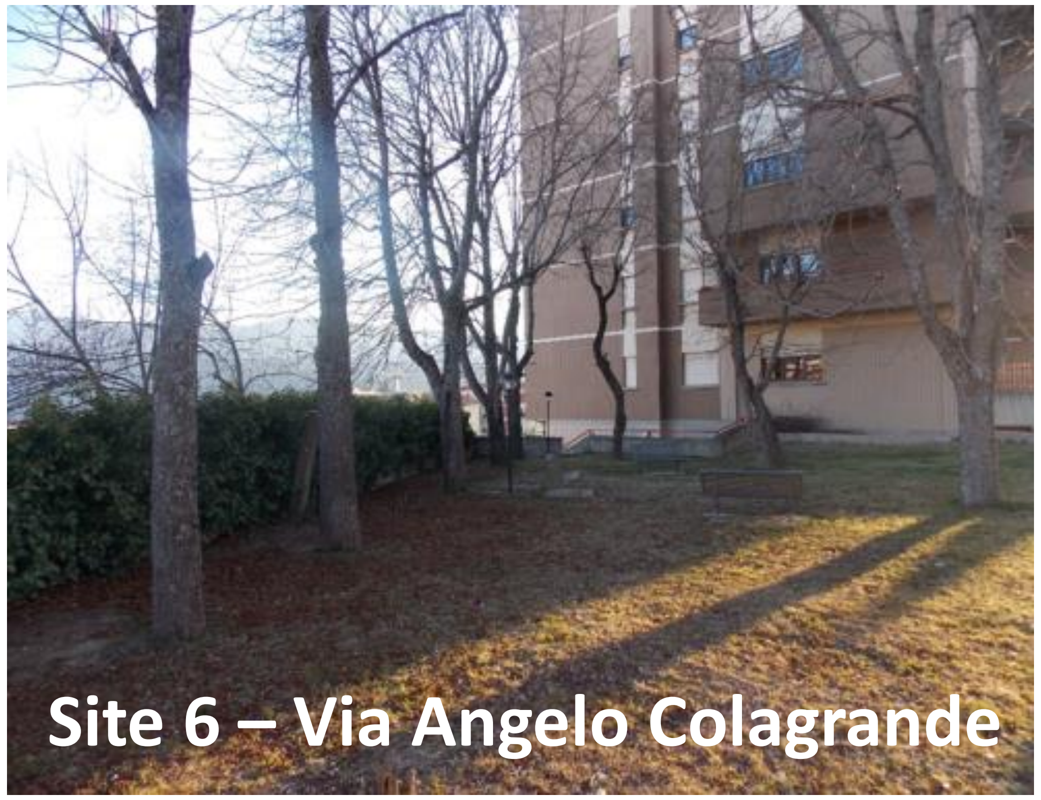
Site 6 – Via Angelo Colagrande

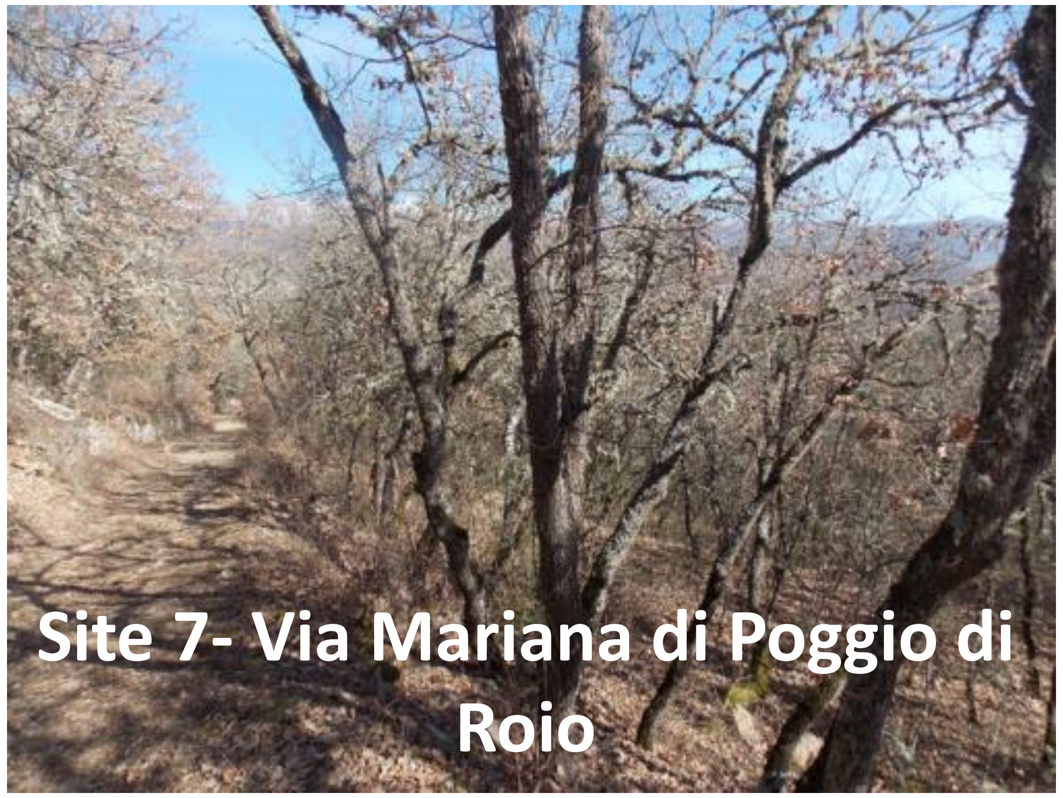
Site 7- Via Mariana di Poggio di Roio

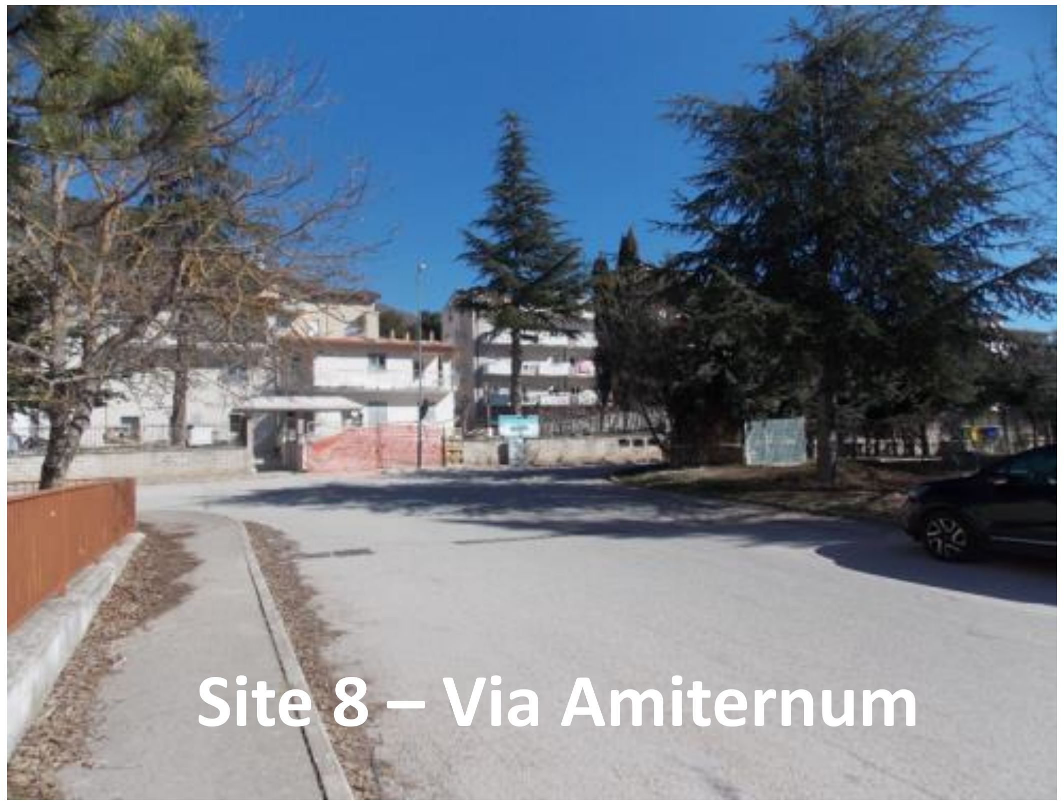
Site 8 – Via Amiternum

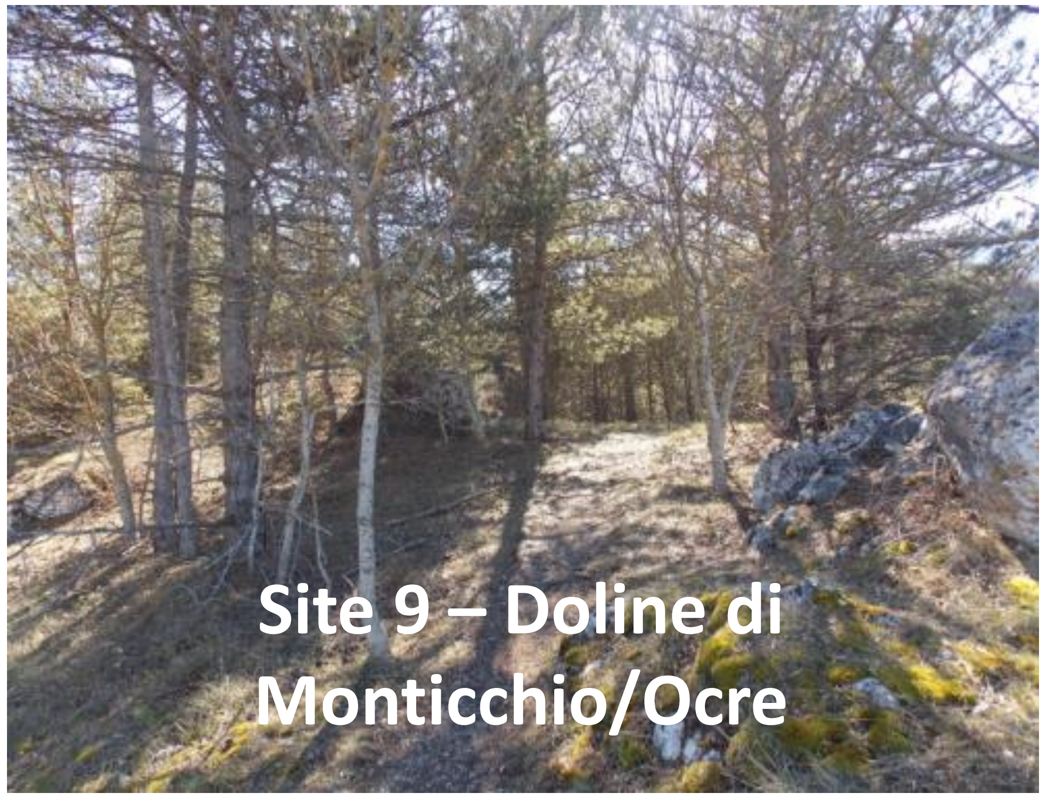
Site 9 – Doline di Monticchio/Ocre
