# Supplementary material for: Use of Lichens to Evaluate the Impact of Post-Earthquake Reconstruction Activities on Air Quality: A Case Study from the City of L’Aquila
Source: Biology (Basel). 2022 Aug 10;11(8):1199. doi: 10.3390/biology11081199 (PMC9405126; doi:10.3390/biology11081199)
Supplement: Supplementary file 1 [file biology-11-01199-s001.zip › SupplementaryMaterials Corrected/Table S1.pdf]

Table S1. Number of lichen species recorded in each of the five quadrats of the vertical grids used to sample epiphytic lichens at nine sites (Sites, numbered from 1 to 9) in the city of L'Aquila (Central Italy). At each site, three trees (numbered from 1 to 3) were sampled, and the grid was placed at the four cardinal points. Site numbers as in Table 1.

Site	Tree	East	South	North	West
1	1	2	1	2	2
1	1	2	1	2	2
1	1	2	1	2	2
1	1	2	1	2	2
1	1	3	2	2	2
1	2	2	1	2	1
1	2	1	1	1	2
1	2	2	1	2	2
1	2	2	1	2	1
1	2	1	1	1	3
1	3	1	0	2	1
1	3	1	0	1	1
1	3	1	0	1	0
1	3	0	1	1	0
1	3	0	1	1	1
3	1	1	2	3	1
3	1	2	2	2	2
3	1	1	2	2	2
3	1	2	2	2	2
3	1	2	1	3	2
3	2	2	2	2	2
3	2	1	2	2	2
3	2	1	2	2	2
3	2	0	2	2	2
3	2	1	1	1	2
3	3	1	1	2	2
3	3	2	1	2	2
3	3	2	0	1	2
3	3	2	0	2	2
3	3	3	1	2	2
4	1	2	2	2	2
4	1	2	2	2	2
4	1	2	2	1	1
4	1	2	1	1	1
4	1	0	0	1	0
4	2	2	2	2	2
4	2	2	2	2	2
4	2	2	0	2	1

4	2	2	1	2	1
4	2	2	1	2	1
4	3	1	2	2	2
4	3	2	2	1	2
4	3	0	2	2	1
4	3	2	2	1	2
4	3	2	1	1	1
5	1	1	2	2	2
5	1	1	1	1	2
5	1	2	1	2	1
5	1	1	2	1	2
5	1	1	1	2	1
5	2	2	1	3	2
5	2	2	0	2	2
5	2	1	0	3	2
5	2	2	1	2	2
5	2	2	0	3	2
5	3	2	2	2	3
5	3	2	2	2	2
5	3	2	2	1	2
5	3	2	1	2	2
5	3	2	2	1	2
6	1	1	1	2	2
6	1	2	2	3	2
6	1	2	1	3	2
6	1	2	1	2	2
6	1	2	1	2	3
6	2	1	1	2	2
6	2	2	1	2	2
6	2	2	0	2	2
6	2	1	0	3	2
6	2	2	1	2	2
6	3	2	2	3	2
6	3	3	2	2	2
6	3	1	1	2	2
6	3	2	2	2	1
6	3	2	2	2	2
7	1	4	2	3	3
7	1	3	3	3	2
7	1	4	2	3	2
7	1	3	2	3	1
7	1	4	2	4	3
7	2	4	3	2	2
7	2	3	3	2	3
7	2	4	3	2	2
7	2	2	2	3	2
7	2	3	4	2	2

7	3	2	2	3	3
7	3	2	3	3	2
7	3	3	3	3	2
7	3	4	4	3	3
7	3	3	3	2	2
8	1	1	1	0	1
8	1	1	1	2	1
8	1	0	0	1	1
8	1	1	0	1	0
8	1	0	0	1	0
8	2	1	1	2	1
8	2	1	0	1	2
8	2	2	1	1	1
8	2	1	1	1	1
8	2	1	0	0	1
8	3	1	1	1	2
8	3	2	1	1	1
8	3	1	1	0	1
8	3	1	2	1	1
8	3	0	1	1	1
9	1	2	3	3	3
9	1	2	3	2	2
9	1	4	2	2	2
9	1	3	2	2	3
9	1	1	1	1	1
9	2	3	2	2	1
9	2	3	3	2	3
9	2	4	4	3	3
9	2	4	2	2	2
9	2	3	3	3	3
9	3	4	3	3	3
9	3	3	2	3	3
9	3	3	4	4	3
9	3	4	3	3	2
9	3	2	3	2	2
