# Supplementary material for: Use of Lichens to Evaluate the Impact of Post-Earthquake Reconstruction Activities on Air Quality: A Case Study from the City of L’Aquila
Source: Biology (Basel). 2022 Aug 10;11(8):1199. doi: 10.3390/biology11081199 (PMC9405126; doi:10.3390/biology11081199)
Supplement: Supplementary file 1 [file biology-11-01199-s001.zip › SupplementaryMaterials Corrected/Table S2.pdf]

Table S2. Presence/absence of lichen species recorded at nine sites (Site, numbered from 1 to 9) in the city of L'Aquila (Central Italy). Site numbers as in Table 1.

**Site**	***Xanthoria parietina***	***Physconia distorta***	***Physcia adscendens***	***Evernia prunastri***	***Parmelina tiliacea***	***Melanelixia glabra***	***Pleurosticta acetabulum***
Site 1	1	1	1	0	0	0	0
Site 2	0	0	0	0	0	0	0
Site 3	1	1	1	0	0	0	0
Site 4	1	1	1	0	0	0	0
Site 5	1	1	1	0	1	1	1
Site 6	1	1	1	0	0	0	0
Site 7	1	1	1	1	1	1	1
Site 8	1	1	1	0	0	0	0
Site 9	1	1	1	1	1	1	1
