# Supplementary material for: Use of Lichens to Evaluate the Impact of Post-Earthquake Reconstruction Activities on Air Quality: A Case Study from the City of L’Aquila
Source: Biology (Basel). 2022 Aug 10;11(8):1199. doi: 10.3390/biology11081199 (PMC9405126; doi:10.3390/biology11081199)
Supplement: Supplementary file 1 [file biology-11-01199-s001.zip › SupplementaryMaterials Corrected/Table S3.pdf]

Table S3. Weight percentages of chemical elements found on samples of the lichen *Xanthoria parietina* sampled at five sites along an urban–rural gradient in the city of L'Aquila (Central Italy). Site numbers as in Table 1. Spectra are given in Figure S1.

Site	C	O	K	S	Si	Mg	Al	P	Fe	Cl	Ca	Na	Ti	Br
1	52.0	45.9	0.6	0.2	0.2	0.2	0.3	0.2	0	0.2	0.2	0	0	0
1	47.8	45.9	1.0	0.3	1.4	0.2	0.7	0.3	0.6	0.4	1.4	0	0	0
1	51.8	46.1	0.6	0.3	0.2	0.2	0.2	0.1	0	0.3	0.2	0	0	0
1	41.8	26.8	7.7	0	7.5	1.3	2.3	0	11.9	0	1.7	0	0	0
1	31.3	49.0	0.6	0.3	1.5	0.2	0.9	0.3	0.6	0.3	15.0	0	0	0
1	21.3	37.2	0.4	0	0.8	0	0.3	0	39.2	0.6	0.2	0	0	0
1	23.5	43.3	0.5	0.2	0.5	0.2	0.3	0	0	0	31.4	0	0	0
1	57.3	39.7	0.7	0.3	0.5	0.2	0.4	0.2	0	0.4	0.4	0	0	0
1	58.8	38.8	0.6	0.3	0.1	0.2	0	0.2	0	0.3	0.4	0	0	0.3
1	49.1	41.2	0.8	0.2	3.1	0.3	1.7	0.2	0.9	0.3	2.1	0	0	0
1	26.9	53.3	0.4	0	0.2	0	0.2	0	0	0	18.8	0.3	0	0
4	49.4	49.0	0.5	0.3	0.4	0	0.2	0.2	0	0.2	0	0	0	0
4	45.6	44.9	0.8	0.3	3.9	0.4	1.9	0.3	1.0	0	1	0	0	0
4	45.7	43.1	0.7	0.4	4.2	0.5	2.2	0.4	1.4	0	1.6	0	0	0
4	51.0	46.7	1.5	0	0.8	0	0	0	0	0	0	0	0	0
4	44.7	44.5	2.8	0	2.1	0	0.9	0.5	2.8	0	1.7	0	0	0
4	18.8	50.9	0.3	0.2	0.5	6.5	0.2	0	0	0	22.6	0	0	0
4	42.0	29.7	0.8	0	0.9	0	0	0	26.7	0	0	0	0	0
4	33.4	31.2	1.2	0	1.6	0	0.8	0.4	18.0	0	12.9	0	0.7	0
4	34.8	26.8	8.1	0	3.6	0	0.8	0	23.5	0	0	0	2.4	0
6	58.5	40.0	0	0	1.0	0	0.5	0	0	0	0	0	0	0
6	45.1	43.9	0.8	0	2.9	0	1.0	0	3.5	0	2.9	0	0	0
6	45.1	49.4	0.5	0.3	2.3	0.4	0.8	0	0.7	0	0.5	0	0	0
6	54.5	39.9	0	0	1.4	0	0.5	0	3.7	0	0	0	0	0
6	44.7	36.7	4.9	0	1.2	0	0	0	12.6	0	0	0	0	0
6	15.2	50.6	0.3	0	10.3	8.1	6.8	0	8.2	0	0.4	0	0	0
6	15.3	53.5	1.2	0	21.1	0.8	5.1	0	2.6	0	0.3	0	0.2	0
6	19.9	51.7	0.6	0	22.1	0.4	1.9	0	3.1	0	0.4	0	0	0
6	20.4	55.3	0.2	0	0.6	0	0.2	0	0	0	23.2	0	0	0
8	51.5	47.0	0.3	0.3	0.3	0.3	0.2	0.2	0	0	0	0	0	0
8	48.6	47.4	0.7	0.4	1.1	0.3	0.6	0	0.5	0.2	0.1	0	0	0
8	49.2	46.8	0.5	0.3	1.4	0.2	0.6	0.2	0.6	0	0.3	0	0	0
8	50.7	46.1	0.7	0.6	0.8	0.3	0.4	0	0	0	0.4	0	0	0
8	28.3	43.3	1.0	0	14	1.1	2.5	0	3.2	0	6.5	0.3	0	0
8	41.2	26.4	0.4	0	11	2.4	1.0	0	10.4	0	7.4	0	0	0
8	52.5	42.6	1.4	0.5	1.2	0	0.3	0	0	0.6	1.0	0	0	0
8	32.9	44.1	1.6	0.2	10.7	4.0	3.9	0	1.9	0.1	0.3	0	0.4	0
8	52.2	34.9	0.4	1.7	9.5	0	0.6	0	0.4	0	0.4	0	0	0
8	28.7	46.5	1.7	0.2	11.3	1.6	5.7	0	3.4	0	0.3	0	0.6	0
9	41.7	46.1	0.8	0.2	4.8	0.7	2.2	0	3.2	0	0.2	0	0	0
9	60.1	38.4	0.3	0.7	0.3	0	0.2	0	0	0	0	0	0	0
9	52.8	45.6	0.3	0.2	0.7	0	0.4	0	0	0	0	0	0	0
9	55.2	43.3	0.4	0.2	0.5	0	0.3	0	0	0	0	0	0	0
9	52.7	46.3	0.3	0.2	0.3	0	0.2	0	0	0	0	0	0	0
9	50.9	45.0	0.4	0	1.8	0.2	0.9	0	0.7	0	0	0	0	0
9	51.5	44.8	0.4	0.2	1.6	0.2	0.8	0	0.5	0	0	0	0	0
