# Supplementary figures and images for: Use of Lichens to Evaluate the Impact of Post-Earthquake Reconstruction Activities on Air Quality: A Case Study from the City of L’Aquila
Source: Biology (Basel). 2022 Aug 10;11(8):1199. doi: 10.3390/biology11081199 (PMC9405126; doi:10.3390/biology11081199)

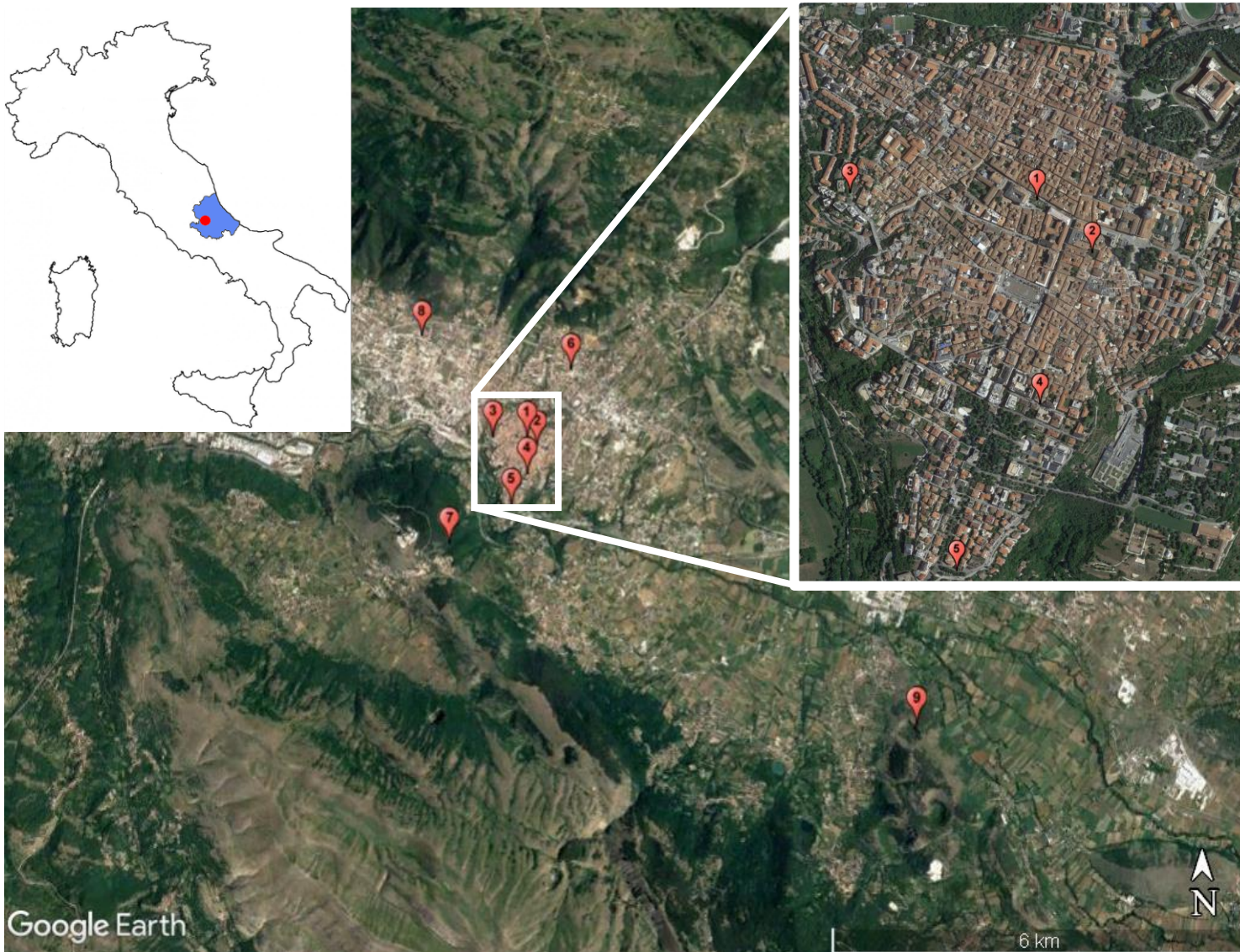

Supplement: Supplementary file 1 [file biology-11-01199-s001.zip › SupplementaryMaterials Corrected/Figure S1.pdf]

Site 4

Sample 4.1

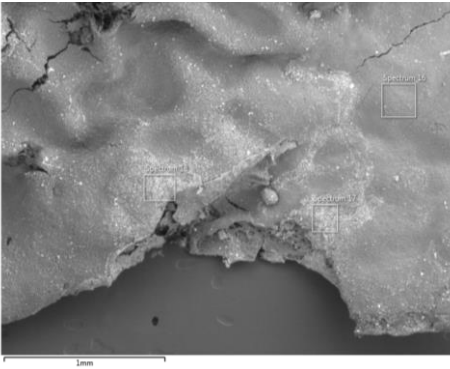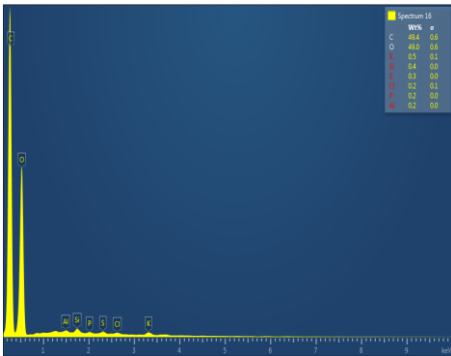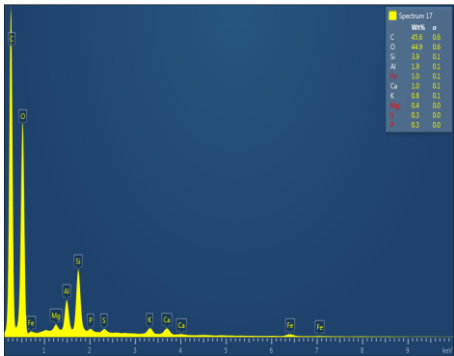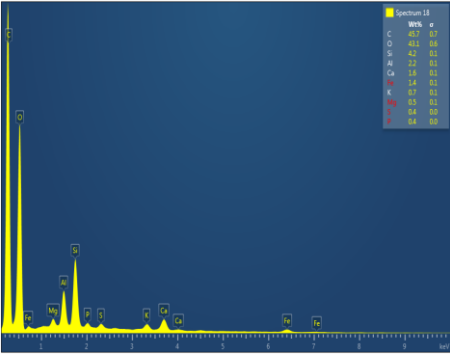

Sample 4.2

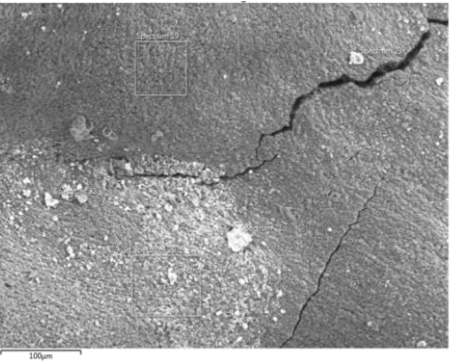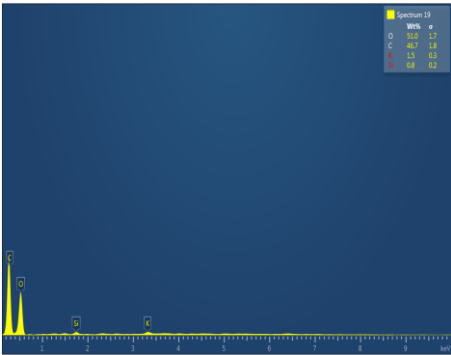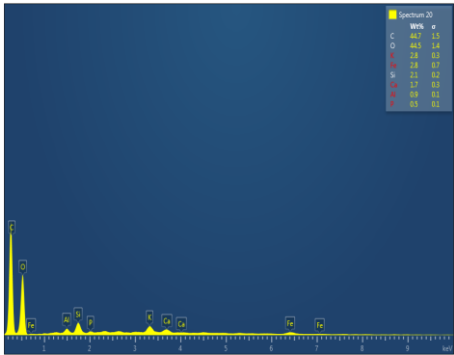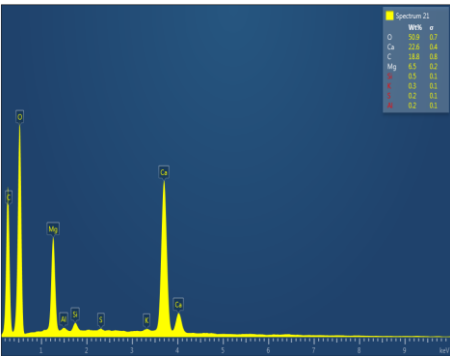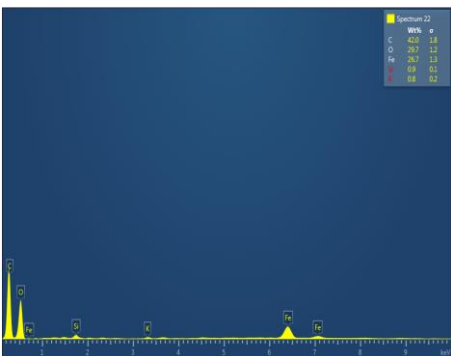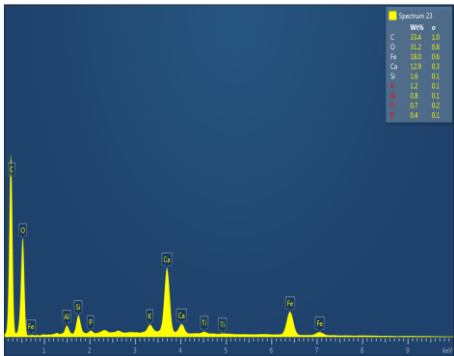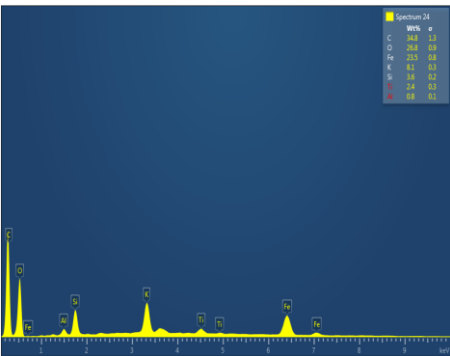

Site 6

Sample 6.1

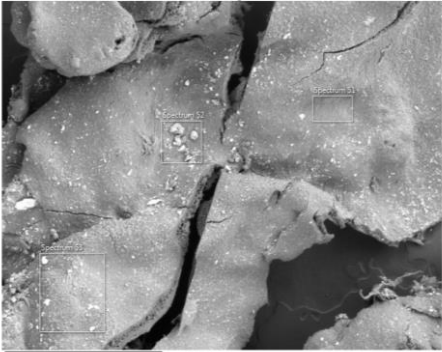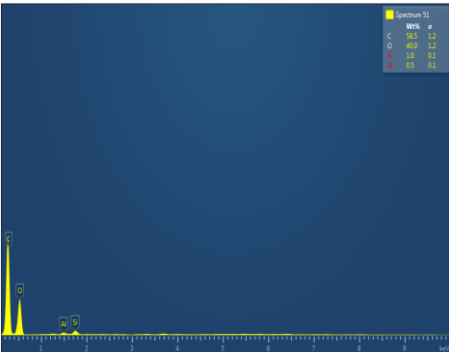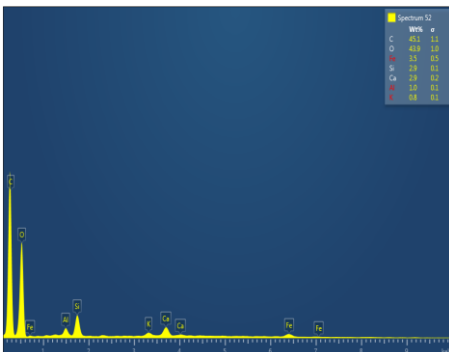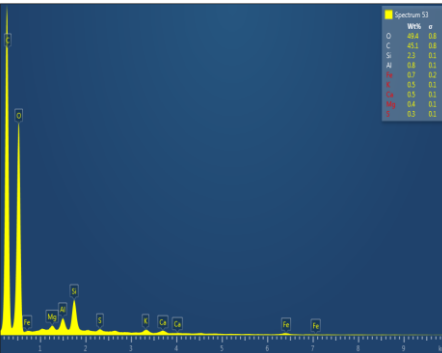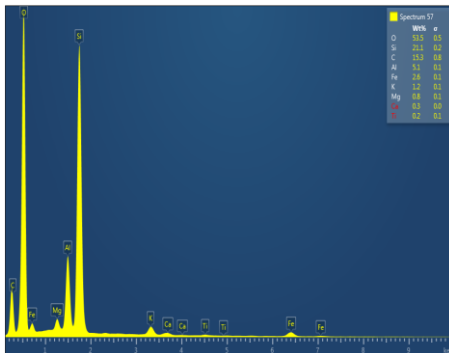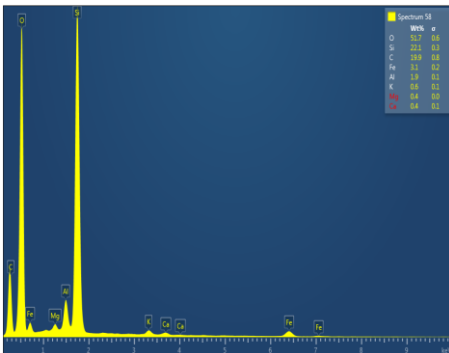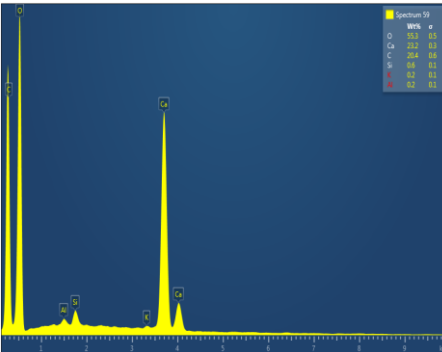

Sample 6.2

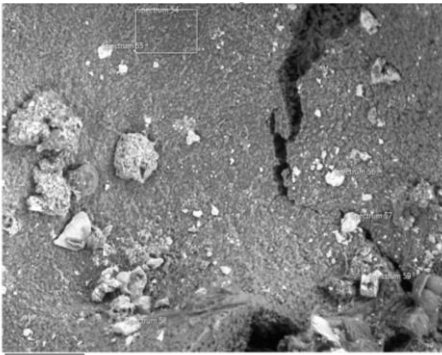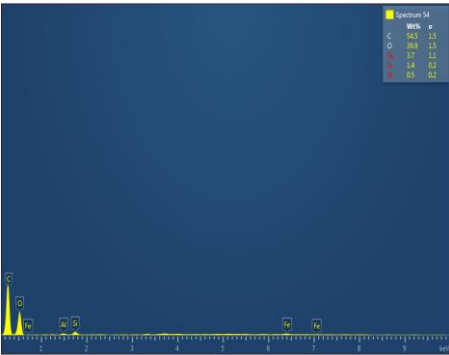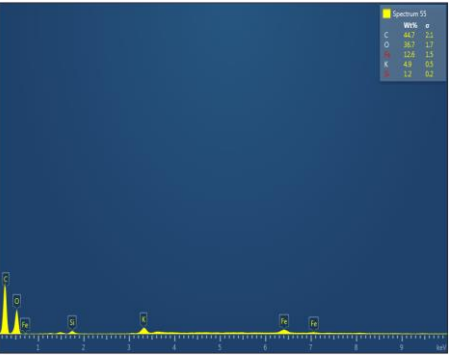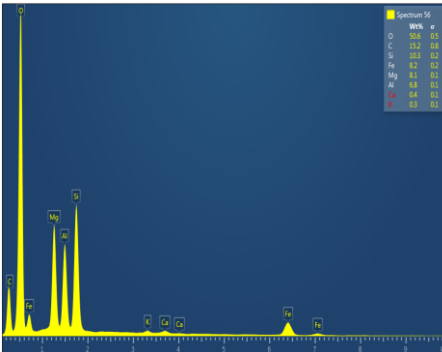

Site 8

Sample 8.1

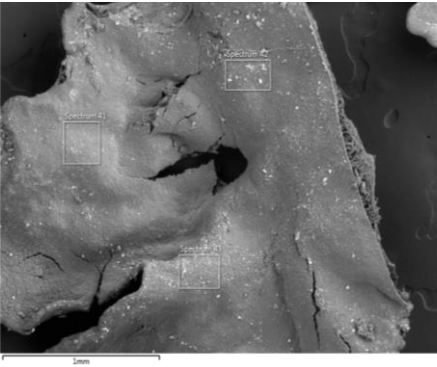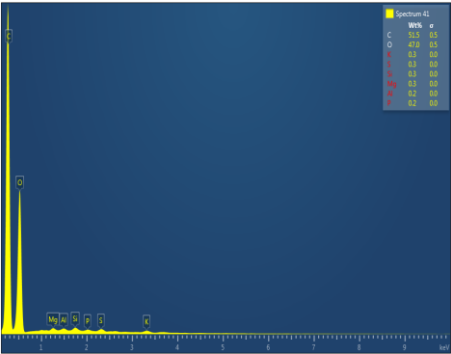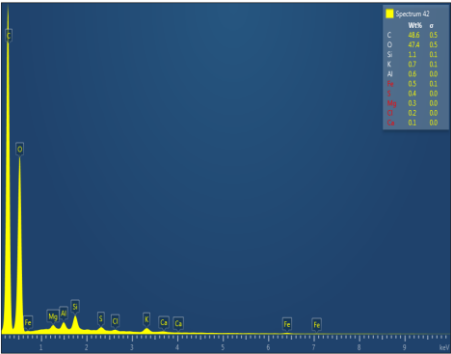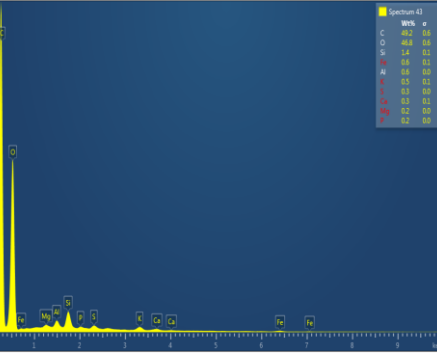

Sample 8.2

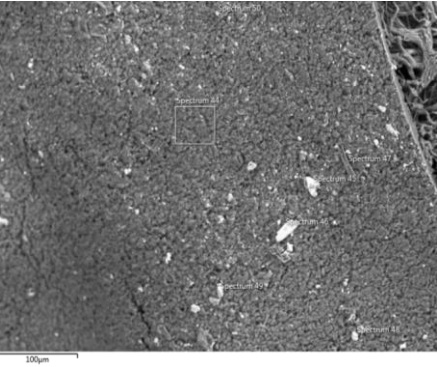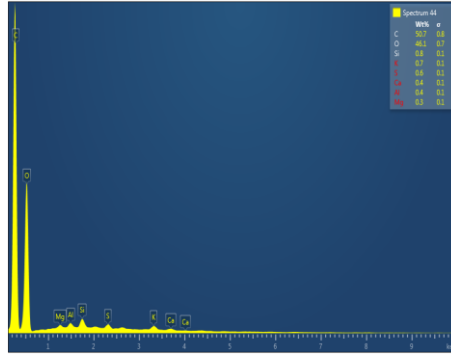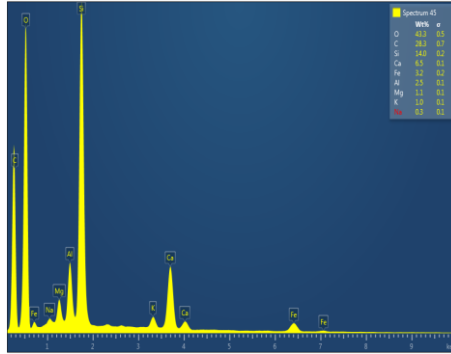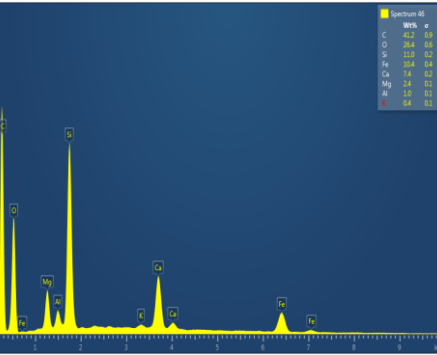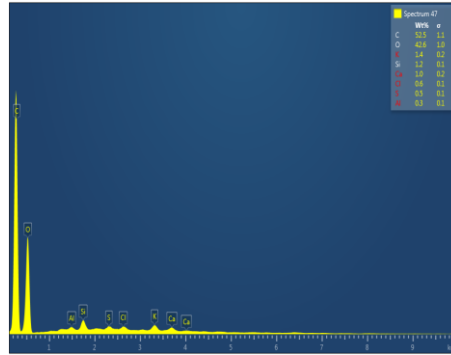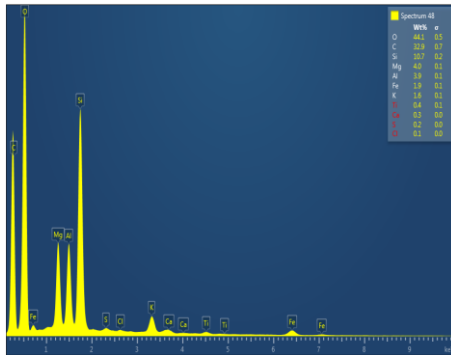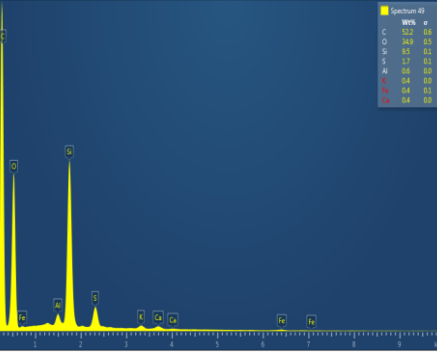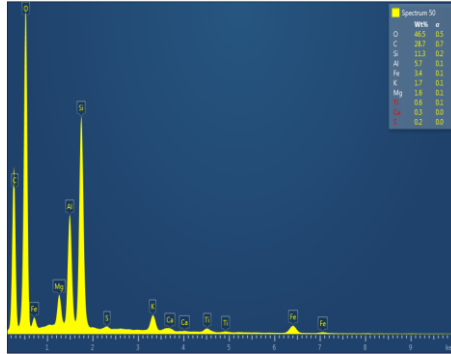

Site 9

Sample 9.1

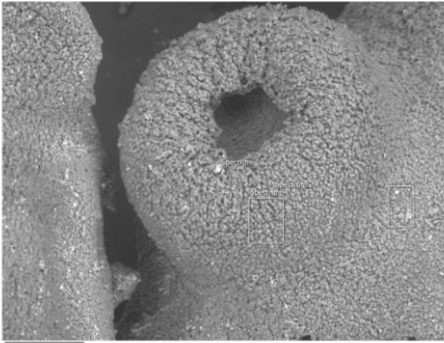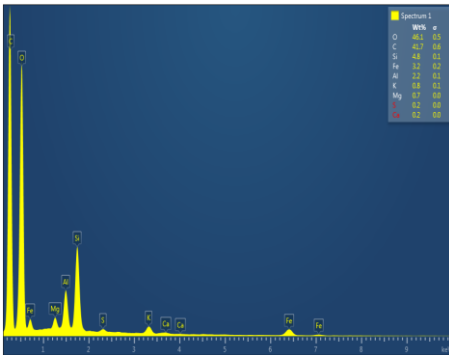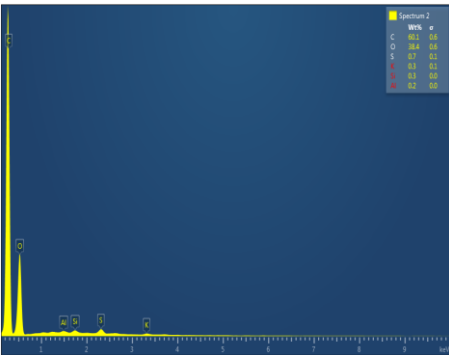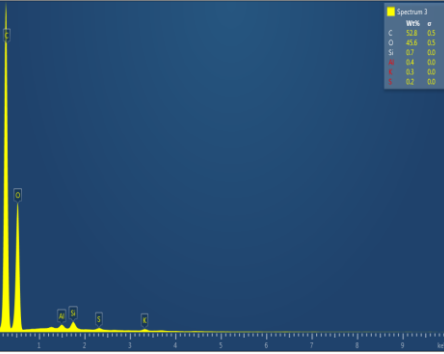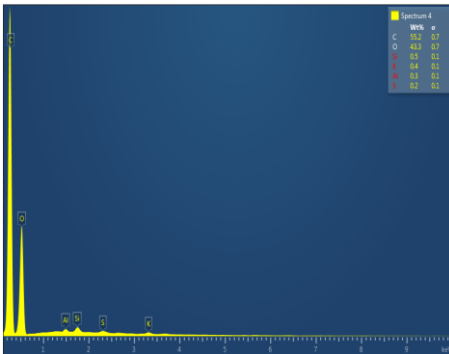

Sample 9.2

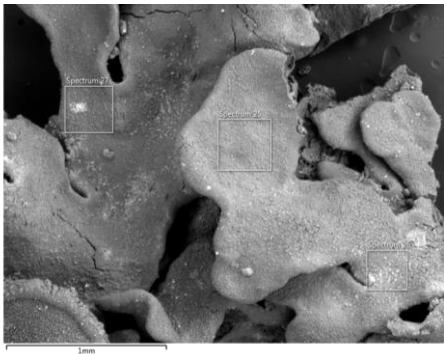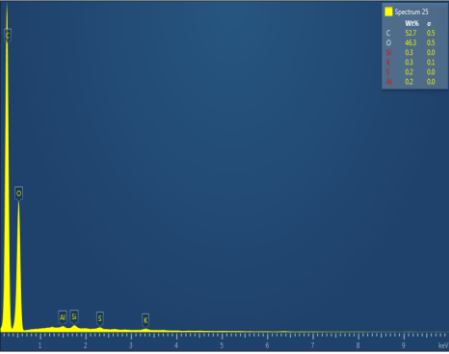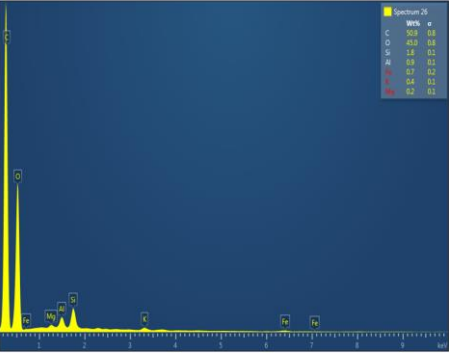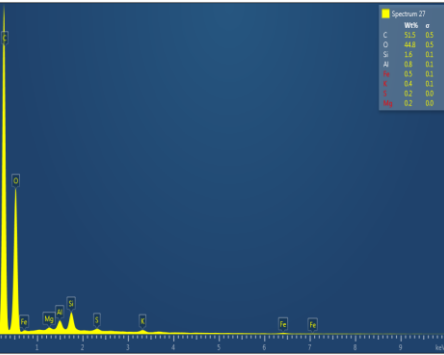

Supplement: Supplementary file 1 [file biology-11-01199-s001.zip › SupplementaryMaterials Corrected/Figure S3.pdf]
